# Tackling tuberculosis patients' internalized social stigma through patient centred care: An intervention study in rural Nicaragua

**DOI:** 10.1186/1471-2458-8-154

**Published:** 2008-05-08

**Authors:** Jean Macq, Alejandro Solis, Guillermo Martinez, Patrick Martiny

**Affiliations:** 1 Ecole de Santé Publique, Université Libre de Bruxelles, Bruxelles, Belgium; 2 Centro de Investigaciones y Estudios de la Salud, Universidad Nacional Autonoma de Nicaragua, Managua, Nicaragua

## Abstract

**Background:**

We report a patient-centered intervention study in 9 municipalities of rural Nicaragua aiming at a reduction of internalized social stigma in new AFB positive tuberculosis (TB) patients diagnosed between March 2004 and July 2005.

**Methods:**

Five out of 9 municipal teams were coached to tailor and introduce patient-centered package. New TB patients were assigned to the intervention group when diagnosed in municipalities implementing effectively at least TB clubs and home visits.

We compared the changes in internalized stigma and TB treatment outcome in intervention and control groups. The internalized stigma was measured through score computed at 15 days and at 2 months of treatment. The treatment results were evaluated through classical TB program indicators. In all municipalities, we emphasized process monitoring to capture contextual factors that could influence package implementation, including stakeholders.

**Results:**

TB clubs and home visits were effectively implemented in 2 municipalities after June 2004 and in 3 municipalities after January 2005. Therefore, 122 patients were included in the intervention group and 146 in the control group. After 15 days, internalized stigma scores were equivalent in both groups. After 2 months, difference between scores was statistically significant, revealing a decreased internalized stigma in the intervention group and not in the control group.

**Conclusion:**

This study provides initial evidences that it is possible to act on TB patients' internalized stigma, in contexts where at least patient centered home visits and TB clubs are successfully implemented. This is important as, indeed, TB care should also focus on the TB patient's wellbeing and not solely on TB epidemics control.

## Background

Tuberculosis (TB) is usually presented as a global public health problem: there were an estimated 9 million new TB cases and 2 million TB deaths in 2004 [1]. TB can also be seen as an individual health problem. TB patients experience psychological, social suffering and their basic rights may be negated [2]. Amongst problems met by TB patients, social stigma is increasingly recognized, often in association with HIV[3]. Social stigma is "an undesirable or discrediting attribute that an individual possesses, thus reducing that individual's status in the eyes of society [4]." It is also "a social process to be understood in relation to the concept of power, domination and difference. It is a process worsening already existing inequalities and exclusions [5]." Two types are usually distinguished: (1) enacted stigma concerns discrimination due to social inferiority, highlighted through people 'running away' from TB patients, while (2) perceived or internalized stigma, used as an outcome in this research, is a sense of inferiority, resulting from fear of enacted stigma, shown by patients hiding their diagnosis from others, or feeling ashamed of having TB [6]. Both types of stigma have been assessed for TB: enacted alone [7], perceived stigma alone [8,9] or both together [10,12].

Some interventions have been reported for their potentiality to reduce tuberculosis social stigma. This has been assessed through qualitative methods, for example in the case of TB clubs [13,14] or involvement of former TB patients in advocacy and mobilization activities [15]. However, no published studies have attempted to compare prospectively changes between a group of TB patients benefiting from these interventions and a control group.

We report hereby an intervention study comparing changes in internalized stigma and treatment outcome between a group of TB patients benefiting from a patient-centered interventions package including at least TB clubs and home visits, and a group control in rural Nicaragua. The basic assumption behind that package design was that, by increasing power-sharing between the health personnel and TB patients (i.e. giving more power to the patient in the health care provider – patient interaction), TB patients' internalized stigma would decrease.

## Methods

In this section, we describe the setting, the population, the definition of control and intervention groups, and the data collection. Data collection was not limited to outcome measurement. We emphasized comprehensive documentation of process before and during interventions package implementation. Indeed, we wanted to capture contextual factors (i.e. those factors external to the intervention package itself, including stakeholder's characteristics) that could influence package implementation.

### Setting and population

Nicaragua is the second poorest country in America. First-line government health services (FLGHS) consist of health posts and health centers. They are managed by a Ministry of Health (MOH) team at the municipal level, under the regional level MOH authorities. TB care is delivered through a MOH program, strongly organized at the FLGHS level. It ensures the availability and quality of free sputum examination in government health laboratories and free TB treatment provided exclusively in FLGHS. In each health center, a nurse is usually responsible for the TB patients care.

This intervention research project involved different stakeholders: (1) it was conceived by the authors of this paper, (2) adapted for its operational design by national TB program coordinator, and MOH team at the municipal and regional level; (3) implemented by MOH teams at municipal level for the operational component and by the authors of this paper for the research component; and (4) it benefited from financial and other supports, for the research component, from Damian foundation project in Nicaragua and Global Fund administrating institution in Nicaragua (NICASALUD).

This research project included 9 municipalities of rural Nicaragua, selected by the MOH national TB program coordinator because of their need to be strengthened for the local management of the care to TB patients. Strengthening local management of the TB program was initially mainly foreseen through operational analysis exercise, explained here bellow.

It targeted new AFB positive TB patients diagnosed between March 2004 and July 2005. Table 1 presents the population, health services and TB diagnosis data for 2003.

**Table 1:** general characteristics of the municipalities (2003 values)

2003 values	Population (proportion living at less than 5 kms from a health facility)	N° consultation/inhabitant and by year	No° of new AFB+ TB cases (N° TB cases diagnosed per 100000 inhabitants)	Number of abandons (proportion of patients starting their treatment)
Siuna	76099 (90%)	0.85	41 (81)	9 (10.7%)
Bluefield	49331 (23%)	0.8	18 (36)	1 (5%)
Waslala	45014 (80%)	2.1	34 (89)	13 (14.3%)
San Jose de Bocay	33635 (90%)	2.3	17 (50)	5 (28%)
La Dalia	48553 (53,5%)	1.42	17 (35)	1 (6%)
Nueva Guinea	117097 (80,3%)	0,42	14 (13)	0 (0%)
El Rama	50 038 (57%)	0.6	22 (43)	2 (10%)
Juigalpa	61234 (80.9%)	1.6	19 (31)	3 (16%)
Wiwili	49443 (-)	1.68	25 (50)	1 (4%)

### The process before implementing the intervention

The process before implementing the intervention covered 5 out of 9 municipalities: Siuna, Bluefield, Waslala, San Jose de Bocay and Nueva Guinea. It was planned in 3 phases: an operational analysis, a qualitative exploration with the design of an intervention package thereafter introduced in the municipalities.

During the first phase, operational analysis was performed. This is a method for identifying problems in the care process for TB patients. It is done through the use of a simple step-by-step model, the operational model. It is based on the passive detection of AFB-positive cases and the main steps that a patient must go through from the first symptoms and up to TB recovery [16]. It makes a distinction between technical (i.e., biomedical) problems, operational (i.e., organization of health services) and social (i.e., behaviour of specific actors) problems. In Nicaragua, the expected benefit from the operational analysis was to improve the abilities of local health personnel to analyze TB patients' care and, thereby, improve the relevance of interventions on TB patients' care. The operational model was meant to be used as part of a "problem-solving" or "managerial" process.

During the second phase, a qualitative exploration of the social stigma meanings and determinants was used as an input for the design of an intervention package in order to reduce internalized stigma.

During the third phase, feedback of the exploration, training on self-esteem and tailoring the intervention package through negotiation with stakeholders were planned.

### The interventions package to act on TB stigma

We initially designed a set of three types of interventions for internalized stigma reduction. Firstly, we wanted to strengthen TB patients through TB clubs taking the form of self-help groups. Secondly, we wanted health personnel to better know TB patients and their realities through performing patient centered home visits and case discussion centered on the problems experienced by TB patients. The objective of the patient centered home visit was to know better the social network of the patient, identify strength and weaknesses of the network, and plan activities to support the patient. Health professionals were expected to listen to the relatives and neighbours of the patients, identify their feelings towards the patient (their fear, disgust, support, etc), and plan with the social network activities to support the patient during his treatment. A specific guide was conceived to assist health care providers. Thirdly, we planned a revised DOT provision through the involvement of a supporter at the first consultation or through reviewing the patient pathway by decreasing unnecessary isolation and decentralizing care where possible.

Of the three types of initially designed interventions, at least TB clubs and patient centered home visits were implemented in the intervention municipalities.

All these interventions were conceived in their implementation with the full participation of MOH authorities. Furthermore, TB clubs had been included in the 2005 Global Fund grant for Nicaragua although it was initially piloted in the intervention municipios by this research project.

### Definition and characterization of the control and intervention groups

We allocated patients in two groups: intervention and control. TB patients were included in the intervention group if diagnosed in a municipality organizing at least the two most consistent interventions of the package: TB clubs and home visits. Others were allocated to control group.

We compared both groups in relation to basic socio-economic indicators: age, gender, declared income, literacy, and distance from the TB treatment center. Additionally, we compared their self-esteem, using the Rosenberg scale [17], at the start of the treatment. Indeed, it is recognized that self-esteem is a strong determinant of internalized stigma.

### Implementation process monitoring

Researchers performed six monitoring visits between February 2004 and December 2005 in each of the five municipalities where interventions package was introduced. During each visit, they documented the progress in implementing interventions and possible municipal health team's initiatives. They performed care process observations and they systematically interviewed various local stakeholders (patients, nurses in charge of TB patients, members of the municipal health team).

### Outcome measurement

Outcomes of this study were TB treatment outcome and TB internalized social stigma.

TB treatment outcome classical indicators were computed with data from the municipal TB register crosschecked with the TB patient cards.

The internalized social stigma was measured through a scale that has been largely inspired by the scale developed by Boyd Ritsher in the context of mental illnesses [18,19]. It includes 10 statements (see Table 2), has been pre-tested [20] and has a relatively good internal consistency (Cronbach alpha = 0,7). It measures four sub-dimensions: alienation, perceived discrimination, stereotypes endorsement and social withdrawal. The scale was applied to the same TB patients at various stages of their treatment (after 15 days, after 2 months), in order to measure the change in their perceptions as a consequence of the care process and exposure to the intervention package. Informed consent was sought before applying the scale. Confidentiality was stressed throughout the research process.

**Table 2:** Proportion of TB patients agreeing with the 10 statements of the stigma scale in control and intervention groups, after 15 days and 2 months of treatment

Statement	Time since starting TB treatment	Proportion of patients that agrees fully with the statement	
		
		Control n = 146 19 missing values	Intervention n = 122 14 missing values
1. I don't have anybody to help me solving economical, familial or sentimental problems	After 15 days	40.00%	35.50%
	After 2 months	46.20%	17.60%
2. People that are not "phtisics" with a lot of cough can't understand my problems	After 15 days	49.60%	55.10%
	After 2 months	45.30%	38.00%
3. I feel ashamed to have tuberculosis	After 15 days	50.40%	46.20%
	After 2 months	45.30%	17.60%
4. We, TB patients, are contagious	After 15 days	55.70%	32.70%
	After 2 months	51.30%	20.40%
5. People do not consider me and don't listen to me because I have tuberculosis	After 15 days	44.30%	35.80%
	After 2 months	42.70%	19.40%
6. People treat me with pity because I have tuberculosis	After 15 days	52.20%	37.40%
	After 2 months	47.00%	16.70%
7. People don't get close to me because I am phtisic, with my lungs damaged and a lot of cough.	After 15 days	43.50%	32.70%
	After 2 months	42.70%	15.70%
8. I am not looking for new relations because I am phtisic, with my lungs damaged and a lot of cough	After 15 days	45.20%	29.90%
	After 2 months	37.60%	13.90%
9. I don't socialize with others because I am afraid to give my point of view and I want to avoid that my family or friends feel ashamed or have problems.	After 15 days	44.30%	33.60%
	After 2 months	39.30%	17.60%
10. I stay away from the people that are not phthisic, with my lungs damaged, to avoid being rejected.	After 15 days	51.30%	36.40%
	After 2 months	47.00%	22.20%

Univariate analysis of TB treatment outcome and internalized TB stigma variable was done using the EPIINFO 2000 program.

### Ethical review

This proposal didn't pass through a formal ethical review. However, it was selected through an independent peer review process to receive financial support from Damian Foundation. It was further on submitted to Nicaragua Ministry of Health authorities for approval.

## Results

A total of 268 AFB positive TB patients were enrolled in the study over the 9 municipalities. We report the process that leads or not to the implementation of intervention, the assignment of TB patients in the control or intervention group, the TB treatment results and the internalized stigma changes.

### The process before implementing interventions

The three phases of the process before implementing the intervention were coached by researchers in Siuna, Bluefield, Waslala, San Jose de Bocay and Nueva Guinea. Upon request of the nurse in charge of TB patients in La Dalia, this municipality was also coached. The municipalities of El Rama, Juigalpa and Wiwili were just visited for data collection.

### Operational analysis

The operational analysis was performed before January 2004 in Bluefield, Siuna, Waslala, San Jose de Bocay and Nueva Guinea. The process and results of this exercise were unequal. In Siuna, decentralization from the health center to the health post was quickly successful. For example, some village health workers transferred sputum samples to the health post and patients from a particular area received their treatment directly from a cured TB patient. Some health posts also provided treatment to TB patients. In Bluefield, the decentralization from health center to health post was difficult at first due to a lack of personnel and resources. However, as of July 2004, some patients received their treatment at the health post or even within their families. In Waslala, the decentralization process was slow but some patients had their treatment supervised by their families after July 2004.

### Context exploration and intervention design

External researchers (i.e. the authors of this paper) performed a qualitative exploration of the meanings and determinants of social stigma in Siuna, Bluefield, Waslala, San Jose de Bocay and Nueva Guinea at the end of 2003 [21].

This consisted in in-depth interviews and focus groups with key stakeholders involved in the TB patients care. The patient centered package design was inspired by this study results.

### Introduction of the intervention package to act on TB stigma

Workshops were organized in each of the 5 municipality during the first semester 2004. Firstly, training on self-esteem was organized by a psychologist in each of the municipality. Secondly, a draft of the interventions package was discussed in each municipality. Each local health team chose amongst the initial package, the interventions to be implemented.

In Bluefield, the whole municipal health team and the TB program regional coordinator participated to the workshop together with nurses from the health center and the health post. They decided to focus on TB patients' clubs and home visits. By the end of the first semester 2004, a meeting was organized by the director of the health center with the whole staff in order to discuss perception and behavior towards TB patients. The regional authorities also organized the broadcast of TB related messages on the local radio.

In Siuna, members of the municipal health team, nurses from the health center and the health posts attended the workshop. Initially, the municipal team decided to implement only home visits but very quickly started also TB patients' clubs.

In Waslala, the whole nursing staff from the municipality participated in the initial workshop but the municipal health director was partly present. The nurse responsible for the care to TB patients in La Dalia, although not supposed to be involved in the study, participated also. The Waslala municipal health team members committed themselves to start home visits and to improve the first consultation process. In July 2004, a follow-up visit was done by researchers and several problems were encountered. A newly appointed medical doctor was performing the first consultation for TB patients. He did not know about the proposed methodology. Concerning home visits, the nurse in charge of TB patients at the municipal level complained of the lack of resources. He performed only two of them, and did not follow the suggested methodology.

In San Jose de Bocay, no authority was present at the initial workshop with the exception of the TB program regional coordinator. The workshop was unprepared and attended only by part of the staff. The decision was taken to decentralize treatment wherever possible. In a second workshop organized in April 2004, few people, different from the first workshop, were present. Many others were involved in the mass vaccination campaign. The follow-up of the February workshop showed little changes and no decentralization. In July 2004, a monitoring visit was done. The municipal director, the nurse and the doctor in charge of TB patients were newly appointed. As a consequence, little changes were observed.

In Nueva Guinea, the workshop mainly discussed the involvement of village health workers in TB suspected cases' referrals. In April 2004, a second workshop was organized. There was confusion as community health workers rather than health staff members were invited to the workshop. It was however decided to organize TB cases discussion and to focus on the reorganization of the TB patient's pathway. During 2004 and 2005, only one case discussion was organized during a monitoring visit. It was very much centered on TB control and not much on the difficulties met by TB patients. The revision of the patient's pathway was not really done because of changes in health staff. In May 2005, TB clubs that had been planned were not organized due to the lack of funds.

### Implementation of the intervention package to act on TB stigma

Three groups of municipalities were identified according to the period home visits and TB clubs were effectively implemented. In the first group, composed of Siuna and Bluefield, it was effectively implemented as of July 2004. In the second group, composed of Waslala, La Dalia and San Jose de Bocay, it was implemented as of January 2005. Finally, home visits and TB clubs were never effectively implemented in the third group composed of Nueva Guinea, Juigalpa, El Rama and Wiwili.

In Bluefield, TB clubs in their new form that is aiming at a maximum of TB patients interactions started operating early 2004. The director and the doctor in charge of epidemiology at the health center were initially present. Home visits were also performed by two health staff members. The regional level proposed a special format for home visits providing information on the TB patients' psychosocial conditions in addition to the usual contact tracing data. In January 2005, the TB clubs were chaired by TB patients themselves. They also decided to appoint an executive board. In Siuna, home visits were done mainly close by the health center. Quickly after, TB clubs started first in the health center upon the initiative of the nurse in charge of TB patients and the doctor responsible of epidemiology. Later on, a TB club executive board constituted of TB patients was set up. In other areas, two cured TB patients took the initiative of starting a club with 5–6 patients. They actively supported the work of the ministry of health in those areas. Another cured TB patient talked on the radio and on TV about tuberculosis and the ways to cure it. Finally, a local NGO decided to support TB clubs meetings.

In Waslala, TB club meetings started to be regularly organized in 2005 with around 20 people present each time. Home visits were done, using the guideline developed in Bluefield. Not all TB patients were systematically visited because some were living too far away. In La Dalia, the nurse in charge of TB patients also started TB patients' club meetings and home visits with the support of the responsible at the regional level. In San Jose de Bocay, a new staff was appointed at the end of 2004. As a consequence, home visits, and TB clubs started. Additionally, the staff of this municipality took the initiative of organizing special counseling for the patients' families, aiming at reducing isolation and increasing support. Finally the decentralization of the diagnosis and treatment at the health post level started for some patients. Some health posts started sputum smear slide preparations for transfer to the health center laboratory.

### Characteristics of TB patients intervention and control groups

Hundred and twenty two AFB positive TB patients were diagnosed in municipalities implementing TB clubs and home visits at that time. They were assigned to the intervention group. The other 146 TB patients included in the study were assigned to the control group (Table 3).

**Table 3:** Number of new AFB+ cases diagnosed by period, by group of *municipalities*, and by control or intervention group

	Municipalities			TB patients	
		
Dates of diagnosis	Group 1 (TB Clubs and home visits started as from 01/07/2004)	Group 2 (TB Clubs and home visits started as from 01/01/2005)	Group 3 (No intervention)	Didn't benefit from TB Clubs and home visits (control group)	Benefited from TB Clubs and home visits (intervention group)
From 01/03/2004 till 01/07/2004	19	23	19	61 (= 19+23+19)	0
From 01/07/2004 till 31/12/2004	40	29	24	53 (= 29+24)	40
From 31/12/2004 till 30/06/2005	35	47	32	32	82 (= 35+47)

Total	94	99	75	146	122

There was no statistically significant difference between the control and intervention groups for socioeconomic variables (Table 4). The mean age in both groups was around 35 years, there were more males than females, and distance to the treatment center averaged 17 kms. Both groups can also be categorized as poor people, with low literacy status and relatively low self-esteem as measured with the Rosenberg scale.

**Table 4:** Characteristics of intervention group compared with control group

	Control n = 146 19 missing values	Intervention n = 122 14 missing values	Stat. sign. (p < 0.05)
Average age	36.2 years	34.4 years	NS
Ratio male/female	1.28	1.28	NS
Average distance between the TB treatment center and patient home	17.7 Km	17.03 Km	NS
Proportion of illiterates	44.9%	40.9%	NS
Proportion without declared income	54.1%	50%	NS
Rosenberg self-esteem scale (between 10 and 40)	22.2	22.6	NS

### Tuberculosis treatment outcomes

The results of the treatment do not show statistically significant difference between the intervention and control groups. Both groups can be considered as having a good outcome: 90% of the TB patients in the control group and 93% of the ones in the intervention group were either cured or completed their treatment; only 5/146 (3%) of the patients in the control group and 4/122 (2%) of the patients in the intervention group abandoned their treatment.

### Internalized stigma scale

Details of the scale are presented on Table 2. We report the proportion of TB patients completely agreeing with the 10 statements after 15 days and 2 months of treatment. In the intervention group, there is an important diminution in the proportion of TB patients agreeing with the 10 statements between 15 days and 2 months of treatment. In the control group, there is no or only slight diminution.

We computed the stigma scale score, attributing 5 points when a TB patient completely agrees with a statement and 1 point when he completely disagrees with it. The total of the scale score amounts therefore between 10 and 50 points. We compared the mean rate for the control group with the mean rate for the intervention group. For both groups, we calculated two internalized stigma scores, one for 15 days of treatment and another one for 2 months of treatment. After 15 days, scores were equivalent in both groups. However, after 2 months, difference between scores was statistically significant, revealing a decreased internalized stigma in the intervention group and not in the control group (Table 5).

**Table 5:** Scale mean score after 15 days and 2 months of treatment in control and intervention group

Scale mean value	Control n = 146 19 missing values	Intervention n = 122 14 missing values	Stat sign
After 15 days	34,6	31,7	p = 0,08
After 2 months	33,1	27,4	p = 0,001
Difference between 2 months and 15 days	1,5	4,3	p = 0,03

## Discussion

**Figure 1:**
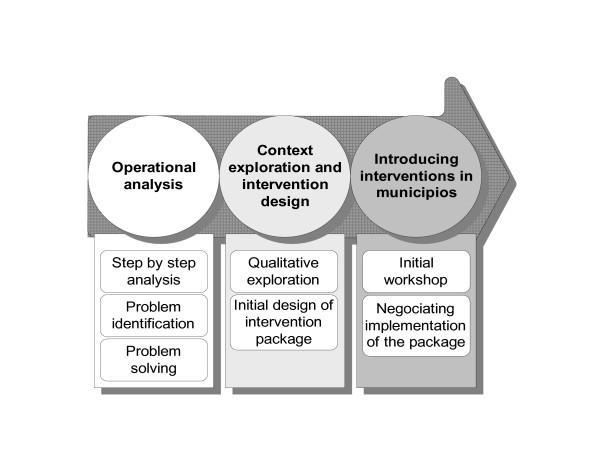
The process before implementing interventions.

Most of the published studies identified the ignorance about tuberculosis, false communities' beliefs and fears as the reasons for tuberculosis social stigma. Suggested solutions are then informing and educating the community [22-24]. We took in this study a different approach. The intervention package tested was centered on the relation between health care providers and the TB patients. The central ingredients of a successful intervention package were the home visits and TB clubs. This was the result of a whole preparation process (see Figure 1). The hypothesis was that by participating in the introduction and the implementation of these core interventions, health personnel would progressively change their attitude towards TB patients, and that TB patients would feel in a stronger position to interact with other stakeholders.

Our study results show that TB patients' internalized stigma is significantly reduced, when a package of interventions including TB clubs and patient centered home visits is successfully implemented. However, the treatment outcome indicators which were already far above WHO targets in both the intervention and control groups didn't change significantly. Reducing only the internalized stigma within a context of good case holding management is important. The need to look at the TB patient's wellbeing, in addition to the TB epidemics control, has indeed been acknowledged in recent international reflections and policies on TB control [25-28].

We should consider this study as an initial step and conclusion concerning outcome measure would need to be confirmed in bigger scale study. Indeed, outcome of this type of intervention should have been probably better analyzed by grouping TB patients by municipios. This would give stronger evidences on the relationship between a given context, stakeholders, intervention package and the outcome. However, given the small scale and the low detection rate of TB patients in the municipios of study, we had to analyze all municipios together. Additionally, this study has been performed in a specific context, i.e. municipios chosen by the national TB coordinator among the least performing in remote area. Conclusion of this study, and mainly external validity, would probably be stronger if similar process would be performed in multiple contexts. Finally, risk of information bias (i.e. patients being aware of the importance of a change between their initial and their second response in the filling of the stigma scale), was prevented in two ways. Firstly, patients were not aware of being part of the intervention or the control group. Secondly, the internalized stigma scale was "self applied" by TB patients with eventually the assistance of a patient's relative, outside of the health care facility and the influence of health care professionals.

These results should not be analyzed mechanically, by trying to link a standardized package of intervention to a given outcome. Acting on TB patients' internalized stigma is a complex issue. Successful interventions depend from the context and the stakeholders and quantitative measures only capture part of the TB patients' feelings.

Firstly, interventions effect cannot be separated from the context. It would be indeed naïve to believe that any type of TB clubs and home visit independently from the context and the stakeholders would improve internalized stigma in TB patients. Indeed in our study, the implementation of the patient centered package has been highly dependent on the context and more specifically on the stakeholders at the local level. Two central stakeholders were critical for a successful intervention package: the TB patients and the nurse in charge of the TB patients. In the first group of municipalities (Siuna and Bluefield), the TB patients played an active role in the TB club activities and other support activities for TB patients, thanks to some individualities' leadership. In the same municipalities, and also in La Dalia, nurses caring for TB patients where highly motivated, knew the community members very well, and were used to working with TB patients, as they have a long lasting experience in such position. In those cases, nurses were supported by authorities: the nurse of Siuna benefited from a dynamic municipal health management team stable during the time of the research; the nurse of Bluefield benefited from the proximity of an enthusiastic TB program responsible at the regional level. Finally, in both Bluefield and Siuna, the work of the nurse responsible of the TB patients, was supported by local NGO's. These findings are congruent with recent policies and experiences emphasizing the pivotal role of frontline health workers in TB care [29-31].

Secondly, quantitative measures only capture part of the TB patients' feelings. In our study, we used a 10 items scale to measure internalized stigma. The presentation of scales to measure TB social stigma in a context of low HIV prevalence has not yet been published in peer reviewed journals. We identified in the "grey" literature five studies that quantified the TB social stigma: Two studies used only one binomial variable [32,33]; three other studies specified sub-dimensions of TB stigma and two used specific scales [10,11]. The scale we used to measure changes in internalized stigma has been applied to TB patients only in our study. Using it in other studies might strengthen its validity. However, as explained in the methodology, it was carefully designed and may be considered as valid for the Nicaraguan context.

Further to its local benefits, this project has been able to influence national policies about the care of TB patients in government health services. The final result of this has been the production of a "*manual de trabajo sobre estigma de la tuberculosis a nivel municipal en Nicaragua*." Part of it has already been used in expanding PATB clubs and home visits in all areas of Nicaragua. It has been discussed and validated by a new commission, the "*Commission Nacional the Apoyo a Personas Afectadas por Tuberculosis*" (CONAPAT) created during, and partly as a consequence of this research project. It involves the main national stakeholders in TB control, i.e. the National TB control program of the Nicaraguan ministry of health, the administrator of the Global Fund (the NGO Nicasalud), the Damian Foundation, a Belgian NGO and the Centro de Investigaciones y Estudios de la Salud (CIES), a public health school.

By its role of coaching, the research team of the CIES has been in a position to support the local implementation of the intervention package, but also to collect information about difficulties and the success in the local processes. This was particularly useful for discussions in the CONAPAT. Indeed, as a consequence of the intertwined process of information collection during coaching activities and discussion of it during CONAPAT meetings, scaling up of tools that were developed in this project was made possible. For example, early 2006, more than 50 municipios created PATB clubs with tools developed by this research.

## Conclusion

Increased attention is given to the TB social stigma and its consequences on TB patients suffering. This study provides initial evidences that it is possible to act on TB patients' internalized stigma, in contexts where at least patient centered home visits and TB clubs are successfully implemented. Outcomes are not dependant of a standardized set of intervention but also of the context, and mainly the capacity of frontline nurse and TB patient to establish a fruitful dialogue. Our findings are relevant particularly, in context where TB control is already effective. Through this paper, we hope to have contributed to promoting an expanding field of research which is highly needed in order to develop valid instruments to measure the stigma, to understand the optimal mix of contextual factors, stakeholders' interaction and interventions' package.

## Competing interests

The authors declare that they have no competing interests.

## Authors' contributions

JM conceived and designed the study; supervised the data collection; analyzed and interpreted the data and wrote the paper. AS conceived and designed the study; supervised the data collection; analyzed and interpreted the data and participated to the writing of the paper. GM conceived and designed the study; supervised the data collection; analyzed and interpreted the data and participated to the writing of the paper. PM conceived and designed the study; supervised the data collection; analyzed and interpreted the data and participated to the writing of the paper. All authors read and approved the final manuscript.

## Pre-publication history

The pre-publication history for this paper can be accessed here:



## References

[B1] WHO (2006). Global tuberculosis control: surveillance, planning, financing. WHO report 2006..

[B2] WHO, WHO (2001). A human right approach to TB.

[B3] Sengupta S (2006). Social impact of tuberculosis in southern Thailand: views from patients, care providers and the community. Int J Tuberc Lung Dis.

[B4] Goffman E (1963). Stigma: notes on the management of spoiled identity.

[B5] Parker R, Aggleton P (2003). HIV and AIDS-related stigma and discrimination: a conceptual framework and implications for action. Soc Sci Med.

[B6] Eastwood SV, Hill PC (2004). A gender-focused qualitative study of barriers to accessing tuberculosis treatment in the Gambia, West Africa. Int J Tuberc Lung Dis.

[B7] Jaramillo E (1998). Pulmonary tuberculosis and health-seeking behaviour: how to get a delayed diagnosis in Cali, Colombia. Tropical Medicine & International Health.

[B8] Johansson E, Long NH, Diwan V (2000). Gender and tuberculosis control Perspectives on health seeking behaviour among men and women in Vietnam. Health Policy.

[B9] Long NH, Johansson E, Diwan VK, Winkvist A (1999). Different tuberculosis in men and women: beliefs from focus groups in Vietnam. Social Science & Medicine.

[B10] Auer C (2003). Strategies for Tuberculosis Control from Experiences in Manila:
The Role of Public-Private Collaboration and of Intermittent Therapy.

[B11] Karim F, Begum I, Islam A, Chowdurry A (2003). Gender barriers to tuberculosis control: Fade-out or in? Key findings and recommendations from the preliminary analysis.

[B12] Kelly P (1999). Isolation and stigma: the experience of patients with active tuberculosis. Journal Of Community Health Nursing.

[B13] Demissie M, Getahun H, Lindtjorn B (2003). Community tuberculosis care through "TB clubs" in rural north Ethiopia. Soc Sci Med.

[B14] He GX, Zhou L, Xu M, Cheng SM (2005). Implementing DOTS strategy through tuberculosis clubs. Int J Tuberc Lung Dis.

[B15] Harries A, Kenyon T, Maher D, Floyd K, Nyarko E, Nkhoma W, WHO  (2001). "Community TB care in Africa", a collaborative project coordinated by WHO. Report on a "lessons learned" meeting in Harare, Zimbabwe, 27-29 September 2000.

[B16] Dujardin B, Kegels G, Buve A, Mercenier P (1997). Editorial: Tuberculosis control: Did the programme fail or did we fail the programme?. Tropical Medicine & International Health.

[B17] Rosenberg M (1965). Society and the adolescent self-image.

[B18] Boyd Ritsher J, Otilingam P, Grajales M (2003). Internalized stigma of mental illness: psychometric properties of a new measure. Psychiatry research.

[B19] Hayward P, Wong G, Bright J, Lam D (2002). Stigma and self-esteem in manic depression: an exploratory study. Journal of Affective Disorders.

[B20] Macq J, Solis A, Martinez G (2005). Assessing stigma of tuberculosis. Psychology, Health & Medicine Journal.

[B21] Macq J, Solis A, Martinez G, Martiny P, Dujardin B (2005). An exploration of the social stigma of tuberculosis in five "municipios" of Nicaragua to reflect on local interventions. Health Policy.

[B22] Alvarez-Gordillo GD, Alvarez-Gordillo JF, Dorantes-Jimenez JE, Halperin-Frisch D (2000). Perceptions and practices of tuberculosis patients and non-adherence to therapy in Chiapas, Mexico. Salud Publica de Mexico.

[B23] Khan A, Walley J, Newell J, Imdad N (2000). Tuberculosis in Pakistan: socio-cultural constraints and opportunities in treatment. Social Science & Medicine.

[B24] Long NH, Johansson E, Diwan VK, Winkvist A (2001). Fear and social isolation as consequences of tuberculosis in VietNam: a gender analysis. Health Policy.

[B25] Lienhardt C, Ogden JA (2004). Tuberculosis control in resource-poor countries: have we reached the limits of the universal paradigm?. Trop Med Int Health.

[B26] Anonymous (2006). A change in direction for tuberculosis control. The Lancet Infectious Diseases.

[B27] Garner P, Volmink J (2006). Families help cure tuberculosis. The Lancet.

[B28] Ogden J, Rangan S, Uplekar M, Porter J, Brugha R, Zwi A, Nyheim D (1999). Shifting the paradigm in tuberculosis control: illustrations from India. International Journal of Tuberculosis and Lung Disease.

[B29] Macq J, Solis A, Martinez G, Dembele M (2005). The frontline TB care providers' supportive systems: findings from three experiences in Central America and West Africa.. International Journal of Tuberculosis and Lung Disease.

[B30] Escott S, Walley J (2005). Listening to those on the frontline: Lessons for community-based tuberculosis programmes from a qualitative study in Swaziland. Social Science & Medicine (1982).

[B31] Jaiswal A, Singh V, Ogden J, Porter J, Sharma PP, Sarin R, Arora VK, Jain RC (2003). Adherence to tuberculosis treatment: lessons from the urban setting of Delhi, India. Trop Med Int Health.

[B32] Ali SS, Rabbani F, Siddiqui UN, Zaidi AH, Sophie A, Virani SJ, Younus NA (2003). Tuberculosis: do we know enough? A study of patients and their families in an out-patient hospital setting in Karachi, Pakistan. The International Journal Of Tuberculosis And Lung Disease: The Official Journal Of The International Union Against Tuberculosis And Lung Disease.

[B33] Cambanis A, Yassin MA, Ramsay A, Bertel Squire S, Arbide I, Cuevas LE (2005). Rural poverty and delayed presentation to tuberculosis services in Ethiopia. Trop Med Int Health.

